# Induced abortion - impact on a subsequent pregnancy in first-time mothers: a registry-based study

**DOI:** 10.1186/s12884-016-1109-3

**Published:** 2016-10-24

**Authors:** Susanna Holmlund, Tommi Kauko, Jaakko Matomäki, Miia Tuominen, Juha Mäkinen, Päivi Rautava

**Affiliations:** 1 Department of Public Health, University of Turku, 20014 Turun yliopisto, Finland; 2 Säkylä Main Health Centre, Säkylä, Finland; 3 Turku Clinical Research Centre, Turku University Hospital, Turku, Finland; 4 Department of Biostatistics, University of Turku, Turku, Finland; 5 Health Care Faculty, Turku University of Applied Sciences, Turku, Finland; 6 Department of Obstetrics and Gynecology, University of Turku, Turku, Finland; 7 Department of Obstetrics and Gynecology, Turku University Hospital, Turku, Finland

**Keywords:** Induced abortion, Termination of pregnancy, First-time mother, Pregnancy, Overweight, BMI, Smoking

## Abstract

**Background:**

To date, several studies concerning the effects of induced abortion (IA) on women’s later psychosocial well-being and future delivery complications have been published. However, the lack of reports on woman’s physical well-being during their first full-term pregnancy occurring after IA is what inspired the current study. Here, we evaluate the physical well-being and use of maternity services of first-time mothers with a history of IA.

**Methods:**

Finnish National Birth Registry data from 2008 to 2010 were linked with the Induced Abortion Registry data from 1983 to 2007. After excluding first-time mothers with a history of miscarriage, ectopic pregnancy or delivery, 57,406 mothers were eligible for the study, with 5,167 (9.0 %) having experienced prior IA. Data from the pregnancy follow-up visits were evaluated and compared between IA mothers and primiparous mothers.

**Results:**

Women with IA had higher rates of smoking after the first trimester and were more likely to be overweight (body mass index >25 kg/m^2^) than the control group mothers. A higher use of maternity health clinic (MHC) services, thrombosis prophylaxis and participation in a second trimester ultrasound and amniotic fluid sample testing were evident in IA mothers, whereas the likelihood of assisted fertilisation procedure(s) was elevated in the control group. A shorter interpregnancy interval (IPI) seemed to contribute to a late first MHC visit and first trimester serum screening test participation, a higher incidence of placenta samples and an increased presence of preeclampsia and maternal care for poor foetal growth.

**Conclusions:**

IA is associated with being overweight before the subsequent pregnancy and with smoking after the first trimester. More frequent pregnancy follow-up visits in the IA group may be due to greater participation in the placenta sample testing and use of thrombosis prophylaxis. No association between IA and preeclampsia, hypertension, gestational diabetes or preterm premature rupture of membranes was evident in the pregnancy parameters. According to our findings, experiencing IA decreased the need for fertilisation procedures before the next pregnancy when compared to primiparous mothers. Among the IA mothers, the short IPI seemed to contribute to the higher risk for preeclampsia and maternal care for poor foetal growth. However, more research is needed around the IPI before establishing its effect on later pregnancy.

## Background

Induced abortion (IA) is something many women undergo at some point in their lives. According to World Health Organization’s (WHO) statistics, 30 % of all pregnancies in Europe end in termination, with the highest and lowest sub-regional termination rates worldwide being in Eastern and Western Europe at 43 % and 12 %, respectively, per 1,000 fertile women [1]. In Finland, IA during the first trimester is considered to be a common procedure in the public health care system, with the decision to seek termination being made by the pregnant woman (and her partner). According to the National Institute for Health and Welfare [2], in 2013, there were 10,120 IAs (8.7/1,000 fertile women) performed in Finland, with the age group of 20–24 having the highest number of IAs. Socioeconomic background was the most common indication in 91.8 % of IAs performed. Medical procedures for IA were introduced in Finland in 2000; today, they account for approximately 95 % of all terminations performed (with a combination of antiprogestin mifepristone and prostaglandin misoprostol used as termination drugs). Over 92 % of all IAs are performed in the first trimester.

Various studies of the effects of IA on woman’s well-being and later deliveries have been published. In Nordic countries with permissive legislation, society has a neutral attitude toward IA procedures and an effective maternity health care system – the psychosocial consequences of IA seem to be minimal [3–7]. Maternity health clinic (MHC) services [8], aiming to monitor and screen the health and well-being of the mother and the unborn baby, and the data from the follow-up visits, are less studied in regard to the effect of terminating a pregnancy. A lack of consistent results regarding pregnancy parameters documented in MHCs (when preventive methods aiding the future delivery could be systematically applied) and the delivery outcomes in pregnancy after IA (e.g. preterm birth [9–16], low birth weight [10, 11], preterm premature rupture of membranes [16, 17] and preeclampsia [18, 19]) inspired this registry-based study. It analyses a large cohort group of mothers to evaluate the effect of IA and its possible complications first in pregnancy and later in delivery parameters. A pregnancy, whether ending in termination or full-term delivery, is always a physiologically deviant situation for the body. Women with a history of IA have already experienced changes in their pregnancy hormone levels as well as in the functioning of the uterus due to being pregnant before and having experienced either a medically or surgically induced abortion in comparison with primiparous women. However, the physical experience cannot be directly compared with a full-term pregnancy because of the short time period; therefore, only short-term changes are evident in the uterus and hormones compared with a full-term pregnancy. By examining the pregnancy variables between women with a history of IA and primiparous women, we hope to add knowledge about IAs’ effects on pregnancy follow-up parameters and how to better predict the possible deviant situations presenting in perinatal time related to the prior IA. We organised the cases into three groups according to the interpregnancy interval (IPI) and gained information on how the pregnancy parameters related to this time interval. The objective of this study was to evaluate whether the use of MHC services and the results from physical examinations differed between first-time mothers with prior IA and primiparous mothers. Being a rather common procedure in public health care, the impact of IA on a following pregnancy should be reported to determine whether there is a need for extra support for this group of mothers. Due to this study’s focus on women becoming mothers for the first time, the possible confounding factor(s) of physical reactions or changes related to experiencing a previous full-term pregnancy were excluded.

## Methods

The National Birth Registry records from 2008 to 2010 were used, in addition to the Registry for Induced Abortions from 1983 to 2007, to locate women becoming mothers for the first time in 2008–2010 to determine the groups for this study. The termination group (*N* = 5,167) consisted of women becoming mothers for the first time with a history of IA. Women pregnant with their first child and with no prior pregnancy history were chosen for the non-termination group (*N* = 52,239). Women with prior miscarriage, ectopic pregnancy or deliveries were excluded from the study. The data from MHC follow-up visits were attained from the National Birth Registry records, and all of the available follow-up parameters were examined between the two groups. For an unknown reason, the mothers’ occupations were not recorded in the National Birth Registry records in 95.0 % of the cases of IA mothers (*N* = 4,910) and 46.6 % of the cases of non-IA mothers (*N* = 24,338). For this reason, we were forced to exclude occupational status from the background characteristics. Other parameters in the National Birth Registry data were routinely recorded, with no other high rates of missing data.

A blood glucose (BG) tolerance test was diagnosed as pathological if it had one of the following three values: at 0 h, BG ≥5.3 mmol/l; at 1 h, BG ≥10.0 mmol/l; at 2 h, BG ≥8.6 mmol/l. The tolerance test is generally performed at 24–28 pregnancy weeks, but if the risk for gestational diabetes mellitus (DM) is thought to be great (e.g. BMI >35 kg/m^2^, type 2 DM in near family, oral corticosteroid treatment), the tolerance test may be performed during pregnancy weeks 12–16 for the first time and again during pregnancy weeks 24–28 if needed.

### Statistical analysis

Distributions were evaluated with the Shapiro–Wilk test of normality. The group differences in categorical background factors were tested using Pearson’s chi-squared test and logistic regression with Tukey’s multiple comparisons adjustment. The group differences in continuous background factors were tested using an analysis of variance (ANOVA) with Tukey’s multiple comparisons adjustment. Additional multivariable model was used to assess confounding effects with certain background variables. All analyses were conducted with the SAS System for Windows version 9.4 (SAS Institute Inc., Cary, NC, USA).

## Results

The IA mothers were more often aged between 20 and 24 years or over 35 years (*p* < 0.0001), more likely to smoke after the first trimester of the on-going pregnancy (*p* < 0.0001) and less likely cohabiting (*p* < 0.0001) than the non-IA mothers (Table 1). Furthermore, IA mothers were more often overweight than non-IA mothers (*p* < 0.0001). Other than this, there were no significant differences in the background characteristics of the groups.

**Table 1:** Background demographics of the first-time mothers in the IA and non-IA groups at the beginning of the on-going pregnancy

Variable		IA group		Non-IA group		
		*n*	%	*n*	%	*p*
Age	<20 y	466	9.02	4,578	8.76	**<0.0001**
	20–24 y	1,555	30.09	12,416	23.77	
	25–29 y	1,683	32.57	19,477	37.28	
	30–34 y	1,008	19.51	11,850	22.68	
	35–39 y	375	7.26	3,368	6.45	
	≥40 y	80	1.55	550	1.05	
Living area	Urban	3,910	77.47	39,376	77.01	0.2188
	Rural	484	9.59	5,292	10.35	
	Densely populated community	653	12.94	6,461	12.64	
Cohabiting	Yes	4,039	78.40	45,312	86.89	**<0.0001**
	No	661	12.83	3,588	6.88	
Smoking	Non-smoking	3,168	62.33	42,794	83.56	**<0.0001**
	Stopped in first trim	652	12.83	3,562	6.96	
	Smokes after first trim	1,263	24.85	4,856	9.48	
BMI (kg/m^2^)	Underweight (<20)	293	5.67	3,548	6,79	**<0.0001**
	Normalweight (20–25)	3,233	62.57	33,949	64.99	
	Overweight (>25)	1,641	31.76	14,742	28.22	

Statistically significant differences indicated with bolded *p*-values

The IA mothers had more follow-up visits at MHCs (13.4 vs 13.3, *p* = 0.0443) and hospitals (3.2 vs 2.9, *p* < 0.0001) than non-IA mothers, with the overall pregnancy follow-up visits totalling up to 16.6 vs 16.2 visits (*p* < 0.0001), respectively. The IA mothers participated more often in the second trimester ultrasound screening (83.84 % vs 82.44 %, *p* < 0.0112) and the amniotic fluid sample testing (2.44 % vs 1.73 %, *p* < 0.0004), and they used thrombosis prophylaxis (1.24 % vs 0.87 %, *p* < 0.0107) more often during their pregnancy than did the non-IA mothers. Experiencing IA seemed to lessen the likelihood for assisted fertilisation procedure(s) (1.95 % vs 5.14 %, *p* < 0.0001) as well as participation in the first trimester serum screening test (33.44 % vs 34.86 %, *p* < 0.0432) when compared to the non-IA mothers. Pathological BG tests were more common in the IA group, but after adjusting for confounding factors, this difference disappeared. No statistical differences were evident between the groups in terms of hospital care during pregnancy, underuse of MHCs, chorionic villus sample procedures, bleeding, high blood pressure, prematurity, anaemia or antenatal corticosteroid use. After adjusting the analyses for age, cohabiting, smoking and weight, participation in the first trimester serum screening test was no longer significant. Interestingly, underutilisation of MHCs and participating in the BG tolerance test became statistically significant only after adjusting for confounding factors, suggesting that these two factors are more frequent among non-IA mothers than among IA mothers (Table 2).

**Table 2:** Pregnancy parameters of the first-time mothers in the IA and non-IA groups

Pregnancy parameter		IA group		Non-IA group		Univariate	Adjusted		
		Mean (SD)	CI 95 %	Mean (SD)	CI 95 %	*p*	*p*	Difference	95 % CI
First visit to MCH (days pregnant)		64.7 (42.0)	63.5–65.9	64.8 (40.5)	64.4–65.1	0.9169	0.7785	−0.1752	−1.40–1.05
Number of MHC visits		13.4 (3.8)	13.3–13.5	13.3 (3.8)	13.3–13.3	**0.0443**	**0.0179**	**0.1359**	**0.02–0.25**
Number of check-ups at hospital		3.2 (2.9)	3.1–3.2	2.9 (2.6)	2.9–3.0	**<0.0001**	**0.0374**	**0.0802**	**0.00–0.16**
Number of all pregnancy follow-up visits		16.6 (4.8)	16.5–16.7	16.2 (4.6)	16.2–16.3	**<0.0001**	**0.0008**	**0.2282**	**0.10–0.36**
		*n*	%	*n*	%	*p*	*p*	OR	95 % CI
Late first visits to MHC (> h12 + 0)		348	6.74	3,229	6.18	0.1167	0.7491	1.02	0.91–1.15
Under-utilisation of maternity care	0	12	0.24	129	0.25			1.406	0.69–2.88
	1–8	97	1.92	1,249	2.46			0.747	0.60–0.92
	>8	4,930	97.84	49,400	97.29	0.0601	**0.0165**	1	
Blood glucose tolerance test performed		2,319	44.88	23,880	45.71	0.2535	**0.0393**	**0.93**	**0.87–1.00**
Pathological blood sugar tolerance level		505	9.77	4,459	8.54	**0.0025**	0.4104	1.04	0.94–1.16
Participation to first trim serum screening		1,728	33.44	18,208	34.86	**0.0432**	0.0543	0.94	0.88–1.00
Participation to second trim serum screening		148	2.86	1,447	2.77	0.6896	0.7093	1.03	0.87–1.23
Participation to first trim ultrasound screening		3,785	73.25	37,751	72.27	0.1336	0.0593	1.07	1.00–1.14
Participation to second trim ultrasound screening		4,332	83.84	43,065	82.44	**0.0112**	**<0.0001**	**1.20**	**1.11–1.31**
Placenta sample taken		52	1.01	497	0.95	0.7076	0.9427	0.99	0.74–1.33
Amniotic fluid sample taken		126	2.44	903	1.73	**0.0004**	**0.0065**	**1.32**	**1.08–1.61**
Hospital care during pregnancy for…									
Bleeding		20	0.39	249	0.48	0.4538	0.2433	0.76	0.48–1.21
High blood pressure		144	2.79	1,489	2.85	0.8265	0.7239	0.97	0.81–1.16
Prematurity		63	1.22	650	1.24	0.9475	0.6770	0.95	0.73–1.23
Other reason		312	6.04	2,900	5.55	0.1533	0.4688	1.05	0.93–1.18
Received any hospital treatment (for any of the reasons mentioned above)		438	8.48	4,243	8.12	0.3792	0.8956	1.01	0.91–1.12
Fertilisation procedure		101	1.95	2,687	5.14	**<0.0001**	**<0.0001**	**0.42**	**0.34–0.51**
Thrombosis prophylaxis		64	1.24	454	0.87	**0.0107**	**0.0038**	**1.49**	**1.14–1.94**
Anaemia		155	3.00	1,525	2.92	0.7293	0.8982	1.01	0.85–1.20
Antenatal corticosteroid treatment		131	2.54	1,390	2.66	0.6176	0.4189	0.93	0.77–1.12

Statistically significant differences indicated with bolded *p*-values

When comparing the most common diagnosis between the groups, a significant difference was observed in gestational DM, at 5.5 % vs 4.6 % (*p* = 0.0034) in IA and non-IA mothers, respectively (Table 3). After adjusting for confounding demographic factors, the observed difference was no longer evident.

**Table 3:** The 10 most frequent diagnoses of the first-time mothers during the on-going pregnancy between the IA and non-IA groups

Diagnosis according to ICD-10	IA group		Non-IA group		*P*-value	*P*-value		
	Frequency	%	Frequency	%	Chi-square	Adjusted model	OR	95 % CI
O13 Gestational hypertension without significant proteinuria	213	4.12	1,982	3.79	0.2406	0.3845	1.07	0.92–1.24
O14.0 Mild to moderate preeclampsia	79	1.53	856	1.64	0.5524	0.5554	0.93	0.74–1.18
O14.0/1/9 All types of preeclampsia	135	2.61	1,458	2.79	0.4567	0.5444	0.95	0.79–1.13
**O24.4 Gestational diabetes mellitus**	285	5.52	2,409	4.61	**0.0034**	0.1418	1.10	0.97–1.26
O24.9 Unspecified diabetes mellitus in pregnancy, childbirth and the puerperium	69	1.34	616	1.18	0.3239	0.8954	1.02	0.79–1.31
O30.0 Multiple gestation	66	1.28	683	1.31	0.8556	0.9129	1.02	0.78–1.31
O32.1 Maternal care for breech presentation	146	2.83	1,654	3.17	0.1802	0.3821	0.93	0.78–1.10
O36.5 Maternal care for known or suspected poor fetal growth	78	1.51	791	1.51	0.9793	0.2455	0.87	0.68–1.10
O42.9 Premature rupture of membranes, unspecified as to length of time between rupture and onset of labour	90	1.74	935	1.79	0.8036	0.5134	1.08	0.86–1.34
O47.0 False labour before 37 completed weeks of gestation	155	3.00	1,427	2.73	0.2614	0.2103	1.12	0.94–1.32
O48 Late pregnancy	72	1.39	694	1.33	0.6979	0.6831	1.05	0.82–1.35

Statistically significant differences indicated with bolded *p*-values

Interpregnancy interval (IPI) categorisation revealed that among women younger than 25, the IPI was often shorter than in older women (Table 4). Only in the 35–39 age group was the IPI similar in >12 months and <6 months when compared to a significantly lower IPI of 6–12 months (7.48 % vs 7.62 % vs 2.81 %, respectively, *p* < 0.0001). Women with an IPI of 6–12 months were more often not cohabiting when compared to women with an IPI <6 months or and IPI >12 months (17.98 % vs 12.50 % vs 12.04 %, respectively, *p* = 0.0116). Weight was not statistically significantly different between the IPI groups, but smoking after the first trimester was clearly more common in IPI <12 months (IPI >12 months 23.17 % vs IPI 6–12 months 34.27 % vs IPI <6 months 30.48 %, *p* = 0.0098).

**Table 4:** Distributions of the interpregnancy interval by significantly differing maternal characteristics

	**<20**		**20−24**		**25−29**		**30−34**		**35−39**		**>40**		
**Age (years)**	**n**	**%**	**n**	**%**	**N**	**%**	**n**	**%**	**n**	**%**	**n**	**%**	**p**
<6 months	26	24.76	33	31.43	27	25.71	10	9.52	8	7.62	1	0.95	<0.0001
6−12 months	49	27.53	65	36.52	40	22.47	19	10.67	5	2.81	0	0	
>12 months	322	7.02	1,363	29.71	1,555	33.89	934	20.36	343	7.48	71	1.55	
	**Yes**		**No**		**N/A**								
**Cohabiting**	**n**	**%**	**n**	**%**	**N**	**%**	**p**						
<6 months	84	80.77	13	12.50	7	6.73	0.0116						
6−12 months	141	79.21	32	17.98	5	2.81							
>12 months	3,615	78.98	551	12.04	411	8.98							
	**Underweight**		**Normalweight**		**Overweight**								
**Weight**	**n**	**%**	**n**	**%**	**N**	**%**	**p**						
<6 months	7	6.67	76	72.38	22	20.95	0.0601						
6−12 months	13	7.30	114	64.04	51	28.65							
>12 months	229	4.99	2,863	62.40	1,496	32.61							
	**No smoking**		**Stopped in first trim**		**Continued after first trim**		**N/A**						
**Smoking**	**n**	**%**	**n**	**%**	**N**	**%**	**n**	**%**	**p**				
<6 months	64	60.95	8	7.62	32	30.48	1	0.95	0.0098				
6−12 months	99	55.62	16	8.99	61	34.27	2	1.12					
>12 months	2,865	62.45	596	12.99	1,063	23.17	64	1.39					

Table 5 demonstrates the statistically significant differences between the IPI groups. A late first visit to MHC was more common among women with an IPI <12 months when compared to women with an IPI >12 months. Participation in the first trimester serum screening test was higher in IPI >12 months than it was in the other intervals. Placenta samples were taken more often in IPI <12 months when compared with IPI >12 months. Blood glucose tolerance test was performed more often among women with IPI >12 months. Preeclampsia was more frequent in IPI <12 months. Furthermore, poor foetal growth was more common in IPI = 6–12 months compared to other mothers. The number of hospital and pregnancy check-up visits seemed to be greater when the IPI was shorter.

**Table 5:** Interpregnancy intervals by statistically significant study variables

	**Yes**		**No**			
	**n**	**%**	**N**	**%**	**p**	
	**Late first visit to MHC**					
<6 months	10	9.52	95	90.48	<0.0001	
6−12 months	30	16.85	148	83.15		
>12 months	305	6.65	4,283	93.35		
	**Participation to first trim serum screening**					
<6 months	13	12.38	92	87.62	<0.0001	
6−12 months	41	23.03	137	76.97		
>12 months	1605	34.98	2,983	65.02		
	**Placenta sample taken**					
<6 months	2	1.90	103	98.10	0.0003	
6−12 months	7	3.93	171	96.07		
>12 months	41	0.89	4,547	99.11		
	**Blood glucose tolerance test performed**					
<6 months	24	22.86	81	77.14	<0.0001	
6−12 months	51	28.65	127	71.35		
>12 months	2,144	46.73	2,444	53.27		
	**Diagnoses O14.0**					
<6 months	5	4.76	100	95.24	0.0073	
6−12 months	5	2.81	173	97.19		
>12 months	64	1.39	4,524	98.61		
	**Diagnoses 014.0/1/9**					
<6 months	7	6.67	98	93.33	0.0067	
6−12 months	8	4.49	170	95.51		
>12 months	111	2.42	4,477	97.58		
	**Diagnoses O36.5**					
<6 months	2	1.90	103	98.10	0.0153	
6−12 months	7	3.93	171	96.07		
>12 months	61	1.33	4,527	98.67		
	**Number of check-ups at hospital**			**Number of all follow-up visits**		
	**Mean (SD)**	**95 % CI**	**P**	**Mean (SD)**	**95 % CI**	**p**
<6 months	3.7 (0.3)	3.2−4.3	**0.0376**	17.9 (0.1)	16.9−18.8	0.0294
6−12 months	3.5 (0.2)	3.1−3.9		16.8 (0.4)	16.1−17.5	
>12 months	3.1 (0.0)	3.0−3.2		16.6 (0.1)	16.5−16.8	

Diagnoses O14.0: Mild to moderate pre-eclampsia

O14.0/1/9: Mild to moderate pre-eclampsia/Severe pre-eclampsia/Unspecified pre-eclampsia

O36.5: Maternal growth for known or suspected poor fetal growth

## Discussion

It seems that women with a history of IA have a tendency for a higher BMI and are therefore more commonly overweight before the following pregnancy than primiparous women are. Participation in the first trimester serum screening test was initially higher in IA mothers, but this difference disappeared after adjusting the analyses for confounding demographic factors. A higher rate of participation in the second trimester ultrasound screening was still evident after the adjustment, suggesting that perhaps the transition to parenthood becomes stronger in the second trimester of pregnancy. The amniotic fluid sample was taken more often in the IA group, possibly because the mothers in this group were more often over 35 years of age than the mothers in the control group. Thrombosis prophylaxis was more common in IA mothers than in non-IA mothers; this difference was evident even after adjusting the results for demographic factors. No studies suggesting this same association were found in the PubMed/MEDLINE search. This finding is challenging to explain, and the clinical significance of the finding may be minimal. Higher participation in amniotic fluid sample testing and use of thrombosis prophylaxis may have contributed to the higher number of MHC and hospital check-ups in the IA group. Underutilisation of MHC services was more evident in the non-IA group than in the IA group, suggesting that perhaps mothers with prior IA are psychologically more aware of their pregnancy and want to ensure the best possible follow-up for their future offspring.

Fertilisation procedures were reported more often in the primiparous group, indicating that infertility after an IA is not a common problem, which supports review by Lowit et al. [20]. Gestational DM first emerged as being present more often in the IA group. However, after adjusting the analyses for confounding factors, the difference was no longer evident, possibly due to the higher rate of overweight and aged mothers in the IA group. More frequent pathological BG tolerance tests were first evident in IA group, but after adjusting the analyses, this difference disappeared. Participating in the BG tolerance test became statically significant only after the adjustment, indicating that risk factors for gestational DM are more often seen in non-IA mothers compared to IA mothers. Any serious complications during a following pregnancy resulting from a prior IA were not evident in this study.

Short IPI (<12 months) was more common in young mothers, mothers living alone and those who continued to smoke after the first trimester of the on-going pregnancy. Even though we were unable to evaluate the educational level or occupation of the mothers due to poor documentation, these findings of age, habitation and smoking are generally considered to be partial indicators of a low socioeconomic status. Late first visits to MHC services are most common in mothers with IPI = 6–12 months when compared to mothers with IPI <6 months or IPI >12 months. This may contribute to the results of mothers with IPI >6 months participating more often in the first trimester serum screening test than mothers with shorter IPIs, possibly because they have missed the time window for the screening test. More frequent participation in the placenta sample testing in mothers with IPI ≤12 months might be explained by the late first visit and missing the first trimester serum screening test. Therefore, if anything pathological is suspected in the first trimester ultrasound screening test, these mothers are guided to the placenta sample test/amniotic fluid sample test. Presence of preeclampsia among IA mothers did not differ from the non-IA mothers in this study. However, in IA mothers, preeclampsia was more common the shorter the IPI was.. This all contributes to the higher number of hospital and total follow-up visits in mothers with shorter IPIs compared to mothers with longer IPIs. Also, maternal care for known or suspected poor foetal growth was more common IPI = 6–12 months than shorter or longer IPIs. After evaluating the literature [21–23], we suggest that one reason for this could be the higher rate of on-going smoking in short-IPI mothers even after the first trimester of the pregnancy.

According to the 2014 perinatal statistics issued by the National Institute for Health and Welfare [24], the mean BMI for all women giving birth was 24.5 kg/m^2^, with approximately 35 % of the mothers being overweight. In addition, 15.3 % of all parturients smoked during pregnancy, with 46 % of these women stopping smoking during pregnancy. A study conducted from 1989 to 2001 [25] examined the effect of IA on the background factors and pregnancy parameters of all pregnant women in the Kuopio region in Finland. Exclusion criteria included multiple pregnancies and foetal structural anomalies. The study concluded that IAs were associated with a maternal age older than 35 years, unemployment, an unmarried status, low educational level, smoking, alcohol consumption, an overweight condition and chronic illnesses in general, even though the proportions of women with DM or high blood pressure did not vary between the groups. Compared to our study, findings of overweight and smoking during pregnancy in the Kuopio study parallel our findings of IA first-time mothers. These divergent findings may be due to the differences in the IA groups in these studies (our first-time mothers vs. all mothers in the Kuopio study).

The strengths of this study are its reliance on the registry-based parameters of the participants and the fact that we were allowed to combine the National Birth Registry records of all of Finland in the years 2008–2010 with the records from the Induced Abortion Registry in order to determine the absolute value of different parameters examined here. No participants were contacted and no questionnaires were completed, thus excluding personal feelings (positive or negative) concerning IA or maternity health care visits. In addition, we felt that all of the participants were in an equal position regarding MHC follow-up, as none of the mothers had prior deliveries and had therefore not attended MHC follow-ups before. In addition, we could assume that, as first-time mothers, there was no stress of prior offspring contributing to these pregnancy follow-up parameters. It would have been interesting to report the symptoms or feelings the mothers themselves may have experienced during their pregnancy in addition to these registry-based facts.

We conclude that in pregnancy follow-up parameters, there are no significant risks evident regarding the upcoming delivery. Prior findings on IAs’ association with preterm birth [9–12] were not evident in our study in any of the scales routinely monitored in MHCs. In addition, differences in the most common diagnoses were not seen when comparing IA and non-IA mothers. However, short IPI seemed to elevate the risk for certain pregnancy complications (preeclampsia and maternal care for known or suspected poor foetal growth). Interestingly, a more frequent pathological BG tolerance test was evident in the first analysis between the groups (Table 2), indicating a higher incidence of gestational DM that was also seen among the 10 most common diagnoses during the on-going pregnancy (Table 3). However, demographic confounding characteristics affected both of these variables because, after adjusting for confounding factors, neither difference was observed. Our future study will examine whether the trend of these pregnancy follow-up findings will continue to be seen in the delivery parameters and the perinatal period in the same sample of mothers. The importance of participating in MHC follow-ups as scheduled and thus being able to participate in the first trimester serum screening test could be emphasised for all of the mothers during their first visit to the MHC in order to increase participation in this screening test and perhaps decrease the number of amniotic fluid samples taken in the IA group. The IPI was found to affect the next pregnancy follow-up from the onset by increasing the risk for a late first visit to an MHC, complicating the follow-up period and predisposing the mother to additional screening tests and follow-up visits to the hospital. Preeclampsia and poor foetal growth were risk outcomes for short IPI; thus, possible IPI should be inquired about during the first visit to the MHC in the beginning of the pregnancy.

## Conclusion

After examining the data of the first-time mothers covering all of Finland in the years 2008–2010, we suggest that IA may be associated with overweight before the next pregnancy and smoking after the first trimester of the on-going pregnancy. IA mothers tend to use MHC services slightly more than non-IA mothers. No association between induced abortion and preeclampsia, hypertension, gestational diabetes or preterm premature rupture of membranes was evident in our study. Short IPI seems to contribute to late first visits to MHCs, adding extra screening procedures to the pregnancy follow-up and burdening the otherwise well-functioning MHC services. Furthermore, our findings suggest that foetal growth and preeclampsia need to be monitored carefully with first-time mothers with short IPIs after IA, although more thorough research in this subject is needed. Already when performing the IA, it could be beneficial to inform the women about the importance of adequate MHC visits in the possible following pregnancy. In addition to the adverse effects of the IA, it is reassuring to find positive outcomes of the procedure, that is, that IA is protective against secondary infertility.

## Abbreviations

DM: Diabetes mellitus
IA: Induced abortion
IPI: Interpregnancy interval
MHC: Maternity health clinic
